# Presenting Symptoms, Incidence, and Risk Factors of Portal Vein Thrombosis After Laparoscopic Sleeve Gastrectomy

**DOI:** 10.7759/cureus.90851

**Published:** 2025-08-24

**Authors:** Jehad T Almutib, Mohammed A Alsahly, Fahad M Al Jabir, Mohammed K Alsharef, Nawaf M Alzahrani, Ibrahim Al Babtain

**Affiliations:** 1 Intensive Care Unit, King Fahad Medical City, Riyadh, SAU; 2 Family Medicine, King Abdulaziz Medical City Riyadh, Riyadh, SAU; 3 Internal Medicine, King Faisal Specialist Hospital and Research Centre, Riyadh, SAU; 4 College of Medicine, King Saud Bin Abdulaziz University for Health Sciences, Riyadh, SAU; 5 Internal Medicine, King Abdulaziz Medical City Riyadh, Riyadh, SAU; 6 General Surgery, King Abdulaziz Medical City Riyadh, Riyadh, SAU

**Keywords:** associated factors, incidence, laparoscopic sleeve gastrectomy, portal vein thrombosis, signs and symptoms

## Abstract

Background/aim

The rate of reported portal vein thrombosis (PVT) has increased over the past decade, coinciding with the rise in gastric sleeve resections. This study aimed to determine the incidence, risk factors, and presenting symptoms of PVT among patients who underwent laparoscopic sleeve gastrectomy (LSG).

Methods

A retrospective medical chart review was conducted for all adult patients who underwent LSG at King Abdulaziz Medical City (KAMC), Riyadh, Saudi Arabia, between January 2016 and December 2020. Data were collected on patients’ sociodemographic characteristics, presenting symptoms, comorbidities, thromboembolic events, medications, smoking status, PVT incidence and subtypes, onset, and time to diagnosis post-LSG. Statistical tests of association were used to identify risk factors associated with PVT.

Results

A total of 495 patients were included (170 males [34.3%] and 325 females [65.7%]), with a mean age of 38.1 ± 12.4 years. PVT occurred in 15 (3.0%) patients. The median time to onset was 14 days. The most common presenting symptoms were diffuse abdominal pain (46.7%), epigastric pain (33.3%), and nausea/vomiting (20.0%). Additional symptoms included right upper quadrant pain, flank pain, and bleeding from the surgical drain. PVT primarily involved the main portal trunk (66.7%) and intrahepatic branches (53.3%). A history of thromboembolic disease was significantly associated with PVT (13.3% vs. 1.9%; p = 0.038). No significant associations were found between PVT and gender (p = 0.639), age (p = 0.972), comorbidities (p > 0.05), smoking (p = 0.672), or medication use (p > 0.05).

Conclusion

The incidence of PVT in this cohort was 3.0%, higher than what has been reported in previous literature. The most common symptom was diffuse abdominal pain. Due to the non-specific nature of the symptoms, clinicians should maintain a high index of suspicion, especially in patients with a history of thromboembolic disease, to facilitate early diagnosis using noninvasive imaging and timely treatment of this potentially life-threatening complication.

## Introduction

Laparoscopic sleeve gastrectomy (LSG) has become one of the most frequently performed bariatric procedures worldwide. It is recognized for its effectiveness in achieving substantial and sustained weight loss, treating morbid obesity, improving weight-related comorbidities, and enhancing patients’ overall quality of life [1-3]. Despite its benefits, LSG is not without complications. Acute hemorrhage occurs in approximately 1% to 6% of cases, gastric leaks in up to 5%, and intra-abdominal abscesses in around 0.7% [4-6].

Obesity itself is a well-established risk factor for thromboembolic events because of its association with hypercoagulability, endothelial dysfunction, and vascular wall abnormalities [7-9]. One rare but potentially life-threatening thromboembolic complication is portal vein thrombosis (PVT), defined as partial or complete obstruction of the portal venous system by thrombus. Thrombi can involve the intrahepatic portal branches and may also extend caudally into the mesenteric or splenic veins [10]. PVT has been reported following various laparoscopic gastrointestinal procedures, including LSG, with an estimated incidence ranging from 0.3% to 1% [11-14]. Reports of PVT have increased over the last decade, likely reflecting the rising global prevalence of gastric sleeve surgeries [14].

The pathophysiology of PVT following LSG is multifactorial. Contributing factors may include an underlying thrombophilic state, prolonged operative duration, elevated pneumoperitoneum pressures, thermal injury to the gastroepiploic vessels during greater curvature dissection, increased intragastric pressure, insufficient thromboprophylaxis, and delayed postoperative mobilization [15-16]. In several studies, the median time to PVT diagnosis after LSG was approximately 20 days, with patients typically presenting with non-specific symptoms such as abdominal pain and vomiting. In all reported cases, the diagnosis was confirmed by contrast-enhanced computed tomography (CT) [17-18].

Despite the clinical relevance of PVT, there remains a paucity of region-specific data describing its incidence, presenting symptoms, anatomic subtypes, and risk factors following LSG. In Saudi Arabia, where bariatric surgery rates have risen sharply in recent years, understanding this complication is critical for guiding prevention and early detection strategies. Therefore, the present study was conducted to determine the incidence of PVT in patients who underwent LSG at King Abdulaziz Medical City (KAMC), Riyadh, Saudi Arabia, to describe their presenting symptoms and PVT subtypes and identify potential demographic, clinical, and perioperative factors associated with its occurrence. In addition, this study specifically addresses the research question: Does a history of thromboembolic disease significantly increase the risk of PVT after LSG in a Saudi population?

## Materials and methods

This retrospective observational study was conducted at KAMC, a tertiary care center in Riyadh, Saudi Arabia, and focused on assessing the incidence of PVT among patients who underwent LSG. The study also aimed to identify potential risk factors and associated clinical features of PVT in this patient population. A total of 495 patients who had undergone LSG between January 2016 and December 2020 were included in the analysis. Eligible patients were adults aged 18 years and above with complete medical records containing demographic details, comorbidity profiles, medication history, and postoperative follow-up information. Patients were excluded if they had incomplete datasets, underwent bariatric procedures other than LSG, or had a prior documented diagnosis of PVT before surgery.

PVT was defined as a partial or complete occlusion of the portal venous system, which included the portal trunk, intrahepatic branches, superior mesenteric vein, or splenic vein. The diagnosis was confirmed in all cases by contrast-enhanced CT performed at the time of symptom onset, with images interpreted by board-certified radiologists as part of standard clinical care. Data were collected retrospectively from electronic health records and compiled using a structured case report form developed specifically for this study. The information obtained included demographic variables such as age, gender, and body mass index (BMI); comorbidities including hypertension, diabetes mellitus, dyslipidemia, thyroid disorders, ischemic heart disease, bronchial asthma, obstructive sleep apnea, and other documented chronic illnesses; lifestyle factors such as smoking status; history of thromboembolic disorders, including deep vein thrombosis, pulmonary embolism, or other venous thromboembolic events; and detailed medication histories, including the use of antihypertensives, oral hypoglycemics, insulin, anticoagulants, antiplatelets, statins, thyroid replacement therapy, and other relevant medications. Perioperative venous thromboembolism (VTE) prophylaxis protocols at KAMC during the study period typically consisted of subcutaneous low molecular weight heparin (enoxaparin 40 mg once daily) initiated within 12 hours after surgery and continued for 7-10 days, unless contraindicated, with routine encouragement of early ambulation within 6-12 hours after the operation. Outcomes related to PVT included its presence or absence, the specific vascular segments involved, the time of onset following surgery, and the presenting symptoms at diagnosis.

Data abstraction was performed independently by two trained investigators, and any discrepancies were resolved by consensus after reviewing the original source documents. When missing or ambiguous data were identified, operative notes, radiology reports, and laboratory records were consulted; if the missing information could not be confirmed, the variable was coded as missing and excluded from the analysis for that variable. Statistical analysis was carried out using SPSS Statistics Version 25.0 (IBM Corp., Armonk, NY). Continuous variables were summarized as means and standard deviations, while categorical variables were expressed as frequencies and percentages. The chi-square test, or Fisher’s exact test where appropriate, was applied to assess associations between the presence of PVT and various demographic, clinical, and treatment-related factors. A p-value of less than 0.05 was considered statistically significant. In the current analysis, there was a statistically significant association between the incidence of PVT and a history of thromboembolic disorders (p = 0.038). The study was conducted in accordance with the principles of the Declaration of Helsinki and received approval from the Institutional Review Board of KAMC. All patient data were anonymized, and confidentiality was maintained in compliance with institutional and national data protection regulations.

## Results

The study included 495 patients, with 170 (34.3%) males and 325 (65.7%) females. The mean age of the patients was 38.11 ± 12.4 years. Half of the patients (n=249, 50.3%) were in the age group of 30 to 49 years (Table 1).

**Table 1:** Demographic characteristics of patients Values presented as numbers and percentages for categorical variables n, sample size or total number of cases; SD, standard deviation

Characteristic	n (%)/mean ± SD
Gender (n=495)	
Male	170 (34.3)
Female	325 (65.7)
Age groups (n=495)	
10 to 29 years	414 (28.5)
30 to 49 years	249 (50.3)
50 years and above	105 (21.2)
Age (n=495)	38.11 ± 12.4
BMI (n=495)	44.47 ± 6.5

Table 2 shows the proportion of patients with comorbid conditions, smoking, and medications. The most common comorbid conditions were diabetes mellitus, documented in 125 (25.3%), followed by hypertension, documented in 117 (23.6%) patients. Eleven (2.2%) patients had a history of thromboembolic disorders, and 48 (9.7%) patients had a history of smoking. Furthermore, 180 patients (36.4%) were on medications, 56 (11.3%) were on oral hypoglycemic agents, 53 (10.7%) were on statins, 45 (9.1%) were on antihypertensive medications, and the rest were taking various other medicines.

**Table 2:** Comorbidities, history of thromboembolic disorders, smoking, and medications among patients who underwent laparoscopic sleeve gastrectomy Values presented as numbers and percentages for categorical variables N, sample size or total number of cases; K, total number of responses

Factors	n (%)
Comorbidities (n=495) (k=784)	
None	197 (39.8)
Diabetes mellitus	125 (25.3)
Hypertension	117 (23.6)
Dyslipidemia	121 (24.4)
Hypothyroidism	58 (11.7)
Hyperthyroidism	3 (0.6)
Ischemic heart disease	5 (1.0)
Bronchial asthma	48 (9.7)
Osteoarthritis	25 (5.1)
Obstructive sleep apnea	12 (2.4)
Gastroesophageal reflux disease	20 (4.0)
Rheumatoid arthritis	2 (0.4)
Anemia	12 (2.4)
Polycystic ovary disease	2 (0.4)
Others	37 (7.5)
History of thromboembolic disorders (n=495)	
Yes	11 (2.2)
No	484 (97.8)
Smoking (n=495)	
Yes	48 (9.7)
No	447 (90.3)
Medications (n=495) (k=622)	
None	315 (63.8)
Antihypertensive	45 (9.1)
Oral hypoglycemic	56 (11.3)
Insulin	24 (4.9)
Statins	53 (10.7)
Antiplatelet	13 (2.6)
Antidepressant	7 (1.4)
Antiepileptic	3 (0.6)
Thyroid replacement	35 (7.1)
Iron replacement	3 (0.6)
Analgesic	5 (1.0)
Steroid	2 (0.4)
Asthma inhalers	20 (4.0)
Proton pump inhibitors	22 (4.5)
Anticoagulants	12 (2.4)
Other	7 (1.4)

**Figure 1:**
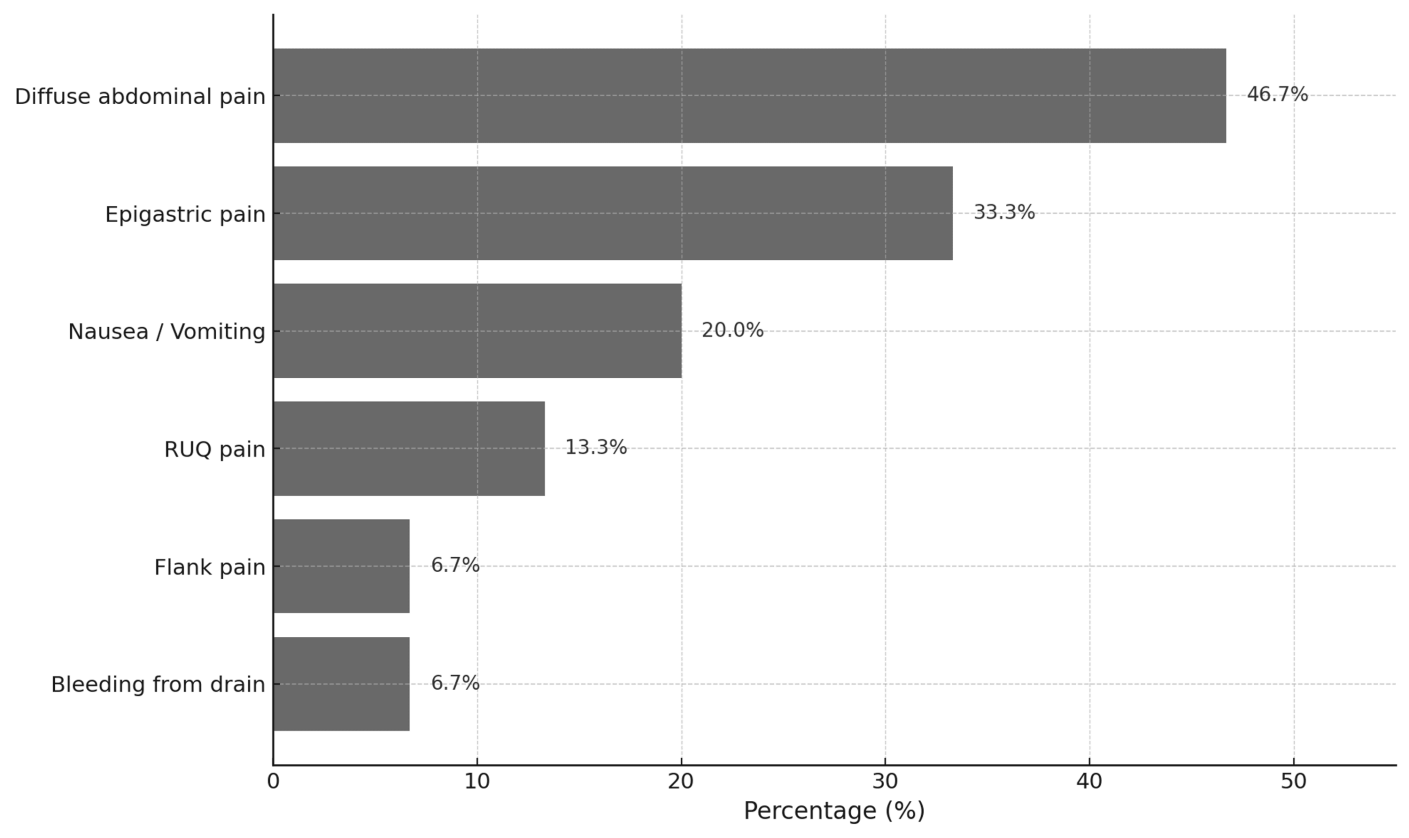
Presenting symptoms of patients with portal vein thrombosis

**Figure 2:**
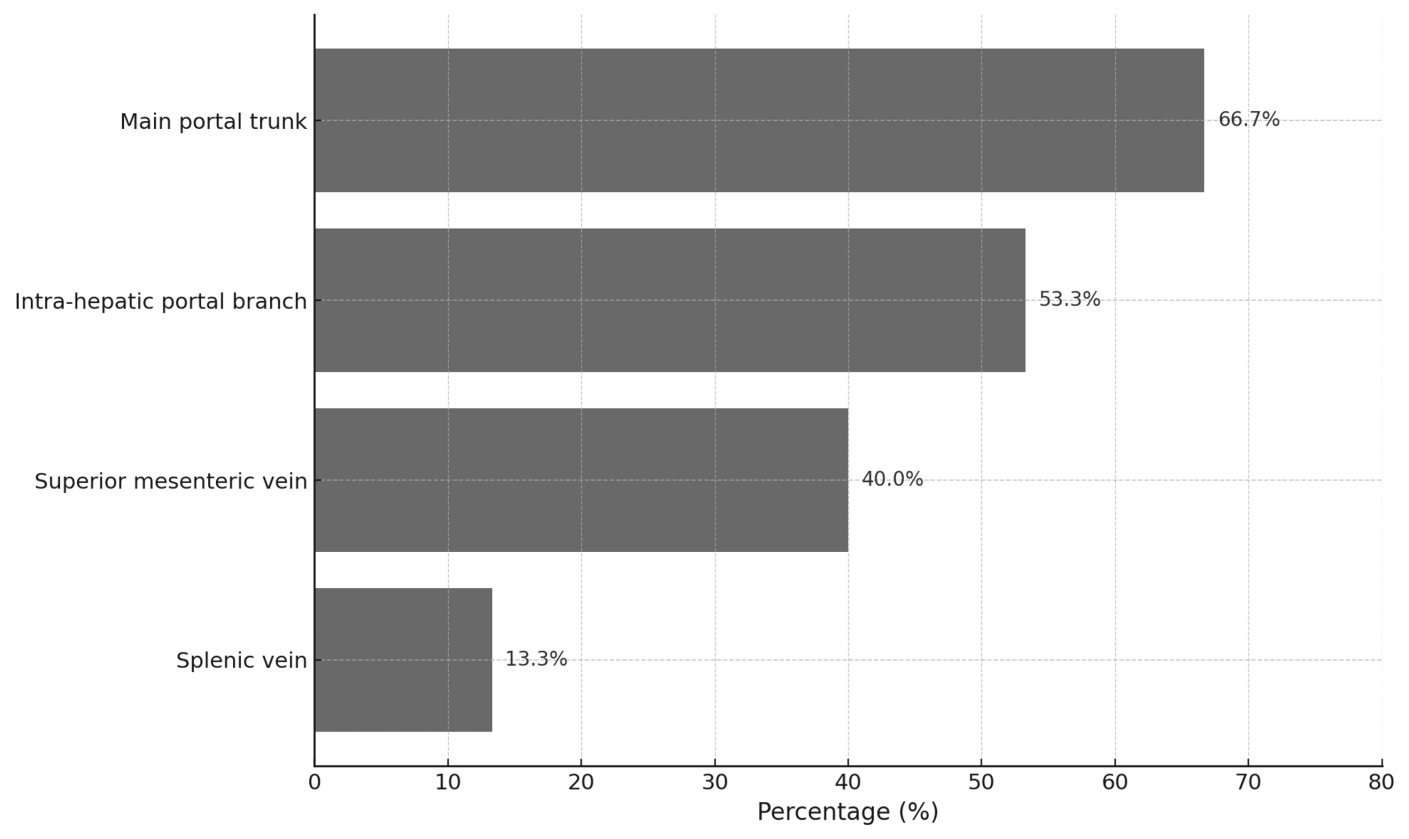
Portal vein thrombosis subtypes in patients

PVT was documented in 15 (3.0%) patients. The median duration of onset of PVT in these patients was 14 days. Of these 15 patients, seven (46.7%) presented with diffuse abdominal pain, five (33.3%) with epigastric pain, and three (20.0%) with nausea and vomiting. Other presenting symptoms included right upper quadrant pain, flank pain, and bleeding from the drain (Figure 1). Among the PVT subtypes, the main portal trunk was noted in 10 (66.7%) of 15 patients and intrahepatic portal branches in eight (53.3%) of cases. Other areas involved are shown in Figure 2.

Test of association between PVT and demographic and expected risk factors showed that patients who had a history of thromboembolic disorder had a significantly greater tendency to have PVT (n=2, 13.3%) compared to those who did not have PVT (n=9, 1.9%) (p=0.038). There were no significant differences in the proportion of patients who had PVT with regard to gender (p=0.639), age groups (p=0.972), presence of comorbid conditions (p>0.05), smoking (p=0.672), and use of different medication (p>0.05). The detailed results of the chi-square test conducted between PVT, and demographic, comorbidities, and expected risk factors are shown in Table 3.

**Table 3:** Association of PVT with demographic factors (gender and age) and expected risk factors (comorbidities, history of thromboembolic disorders, smoking, and medications) *Statistically significant at 0.05 level PVT, portal vein thrombosis; HTN, hypertension; DM, diabetes mellitus; DLP, dyslipidemia; IHD, ischemic heart disease; BA, bronchial asthma; OSA, obstructive sleep apnea; GERD, gastroesophageal reflux disease; RA, rheumatoid arthritis; PCOS, polycystic ovaries syndrome

Variable	PVT, yes, n (%)	PVT, no, n (%)	P-value	χ²
Gender				
Male	6 (40.0)	164 (34.2)	0.847	0.037
Female	9 (60.0)	316 (65.8)	-	-
Age				
10–29 years	4 (26.7)	137 (28.5)	1	0
30–49 years	8 (53.3)	241 (50.2)	-	-
50+ years	3 (20.0)	102 (21.3)	-	-
Comorbidities				
None	8 (53.3)	189 (39.4)	0.277	-
HTN	2 (13.3)	115 (24.0)	0.311	-
DM	4 (26.7)	121 (25.2)	0.899	-
DLP	3 (20.0)	118 (24.6)	0.678	-
Hypothyroidism	2 (13.3)	56 (11.7)	0.846	-
Hyperthyroidism	0 (0.0)	3 (0.6)	0.667	-
IHD	0 (0.0)	5 (1.0)	0.578	-
BA	1 (6.7)	47 (9.8)	0.672	-
OA	0 (0.0)	25 (5.2)	0.209	-
OSA	0 (0.0)	12 (2.5)	0.387	-
GERD	0 (0.0)	20 (4.2)	0.262	-
RA	0 (0.0)	2 (0.4)	0.725	-
Anemia	0 (0.0)	12 (2.5)	0.387	-
PCOS	0 (0.0)	2 (0.4)	0.725	-
Others	0 (0.0)	37 (7.7)	0.124	-
History of thromboembolic disorders				
Yes	2 (13.3)	9 (1.9)	0.038*	4.307
No	13 (86.7)	471 (98.1)	-	-
Smoking				
Yes	1 (6.7)	47 (9.8)	1	0
No	14 (93.3)	433 (90.2)	-	-
Medications				
None	9 (60.0)	306 (63.7)	0.766	-
Antihypertensive	1 (6.7)	44 (9.2)	0.729	-
Oral hypoglycemic	1 (6.7)	55 (11.5)	0.536	-
Insulin	0 (0.0)	24 (5.0)	0.218	-
Statins	3 (20.0)	50 (10.4)	0.283	-
Antiplatelet	1 (6.7)	12 (2.5)	0.4	-
Antidepressant	0 (0.0)	7 (1.5)	0.51	-
Antiepileptic	0 (0.0)	3 (0.6)	0.667	-
Thyroid replacement	1 (6.7)	34 (7.1)	0.95	-
Iron replacement	0 (0.0)	3 (0.6)	0.667	-
Analgesic	0 (0.0)	5 (1.0)	0.578	-
Steroid	0 (0.0)	2 (0.4)	0.725	-
Asthma inhalers	0 (0.0)	20 (4.2)	0.262	-
Proton pump inhibitors	1 (6.7)	21 (4.4)	0.691	-
Anticoagulants	1 (6.7)	11 (2.3)	0.367	-
Other	0 (0.0)	7 (1.5)	0.51	-

## Discussion

PVT is a rare but potentially fatal complication of laparoscopic surgery, including bariatric procedures [19-21]. Previous studies have estimated the incidence of PVT following sleeve gastrectomy to be approximately 1% [20]. In the present study, the incidence was higher at 3.0%, exceeding most previously published rates [14,20]. This difference may reflect variations in patient characteristics, detection practices, perioperative protocols, or other institution-specific factors.

The etiology of PVT after abdominal surgery is multifactorial, involving both systemic and local contributors. Systemic factors include obesity, malignancy, and inherited thrombophilias, while local contributors relate to inflammation and surgical manipulation of the portomesenteric venous system, such as that seen following splenectomy [22]. During laparoscopic procedures, elevated intra-abdominal pressure from pneumoperitoneum and the steep reverse Trendelenburg position can impair portal venous flow, creating a transient prothrombotic state [20,21]. When combined with the hypercoagulable milieu associated with obesity, these factors may further increase PVT risk. Procedural ischemia may also play a role, potentially contributing to portal pyemia and thrombosis. Notably, LSG has been linked to a greater reduction in portal venous peak systolic flow velocity in the early postoperative period compared with other bariatric operations [23].

Patients who develop PVT after bariatric surgery typically present within six weeks of the procedure, often with vague and non-specific symptoms such as abdominal pain and nausea. In our cohort, the median time to diagnosis was 14 days, although one patient was diagnosed as late as 270 days postoperatively [17,18,20]. Vital signs and laboratory parameters are frequently normal, and the diagnosis is generally confirmed by contrast-enhanced CT, which may also reveal intestinal ischemia if the thrombus extends into the superior mesenteric vein [20,21].

In this study, a history of thromboembolic disorders was significantly associated with PVT occurrence. This finding may be explained by underlying inherited or acquired thrombophilic states. For example, protein C or protein S deficiency has been identified as a risk factor for PVT in cirrhotic patients and as a prognostic marker in liver transplantation candidates [24,25]. The surgical technique in LSG, which involves transection of the short gastric veins, could also influence thrombotic risk. Other hereditary thrombophilias, such as factor V Leiden mutation, prothrombin G20210A mutation, antithrombin III deficiency, homozygous MTHFR mutation, and hyperhomocysteinemia, may contribute and warrant consideration [26].

Management of PVT depends on clinical severity and underlying etiology. Patients with signs of intestinal ischemia require urgent surgical or endovascular intervention [21,22], whereas those who are hemodynamically stable should receive prompt anticoagulation. In individuals with systemic prothrombotic conditions, lifelong anticoagulation may be indicated, aiming for portal vein recanalization [27]. Although extended thromboprophylaxis after bariatric surgery is not universally recommended, several studies suggest it may reduce late thromboembolic events; however, the practice remains controversial [28,29].

In our analysis, no significant associations were found between PVT and gender, age, comorbidities, smoking, or medication use, underscoring the challenge of predicting risk based solely on routine clinical factors. Some experts advocate for preoperative thrombophilia screening, particularly in patients with personal or family histories of thromboembolism. Early postoperative ambulation remains a critical preventive measure and should be closely monitored. Patients with known thrombophilic conditions may benefit from extended anticoagulation for 6-12 months, in addition to mechanical measures such as intermittent compression devices and compression stockings, ideally under hematology co-management [28-30].

This study has notable strengths, including its relatively large sample size from a single high-volume tertiary center, which allows for more precise incidence estimates, and its focus on a clinically significant but under-reported complication in a Middle Eastern population. The use of standardized perioperative care protocols enhances internal consistency. Nevertheless, several limitations must be acknowledged. The retrospective design introduces potential selection bias and unmeasured confounding, and causality cannot be inferred from observed associations. The number of PVT cases was relatively small, limiting statistical power for risk factor analysis and increasing the likelihood of type II error. Although PVT diagnosis was confirmed by CT interpreted by board-certified radiologists, no centralized re-review of images was conducted. Thrombophilia testing was not routinely performed, which prevented assessment of inherited prothrombotic disorders. Finally, while the institutional VTE prophylaxis regimen has been described, post-discharge adherence was not systematically documented.

In summary, the higher-than-expected incidence of PVT in this cohort may reflect both patient- and practice-related factors. The significant association with a history of thromboembolic disease highlights the importance of targeted vigilance in this subgroup, although this relationship should be interpreted with caution given the study’s limitations. Future multicenter prospective studies with standardized imaging protocols, comprehensive thrombophilia evaluation, and detailed prophylaxis documentation are warranted to better define at-risk populations and inform preventive strategies.

## Conclusions

In this single-center cohort, the incidence of PVT following LSG was 3.0%, which is higher than most previously reported rates. The most common presenting complaint was diffuse abdominal pain, although symptoms were often non-specific. Because of this ambiguity, clinicians should maintain a high index of suspicion for PVT, particularly in patients with a history of thromboembolic disease, to facilitate timely use of appropriate noninvasive imaging and early initiation of therapy.

Given the retrospective nature of this study, the relatively small number of PVT cases, and the absence of routine thrombophilia testing or systematic documentation of post-discharge prophylaxis adherence, these findings should be interpreted with caution and do not establish causation. Nevertheless, they underscore the need for heightened vigilance, early diagnosis, and prompt management of this potentially life-threatening complication. Future prospective, multicenter studies with standardized diagnostic protocols and comprehensive risk factor evaluation are warranted to confirm these observations and inform evidence-based preventive strategies.
